# Supplementary figures and images for: Appropriateness of the current parasitological control target for hookworm morbidity: A statistical analysis of individual-level data
Source: PLoS Negl Trop Dis. 2022 Jun 28;16(6):e0010279. doi: 10.1371/journal.pntd.0010279 (PMC9239476; doi:10.1371/journal.pntd.0010279)

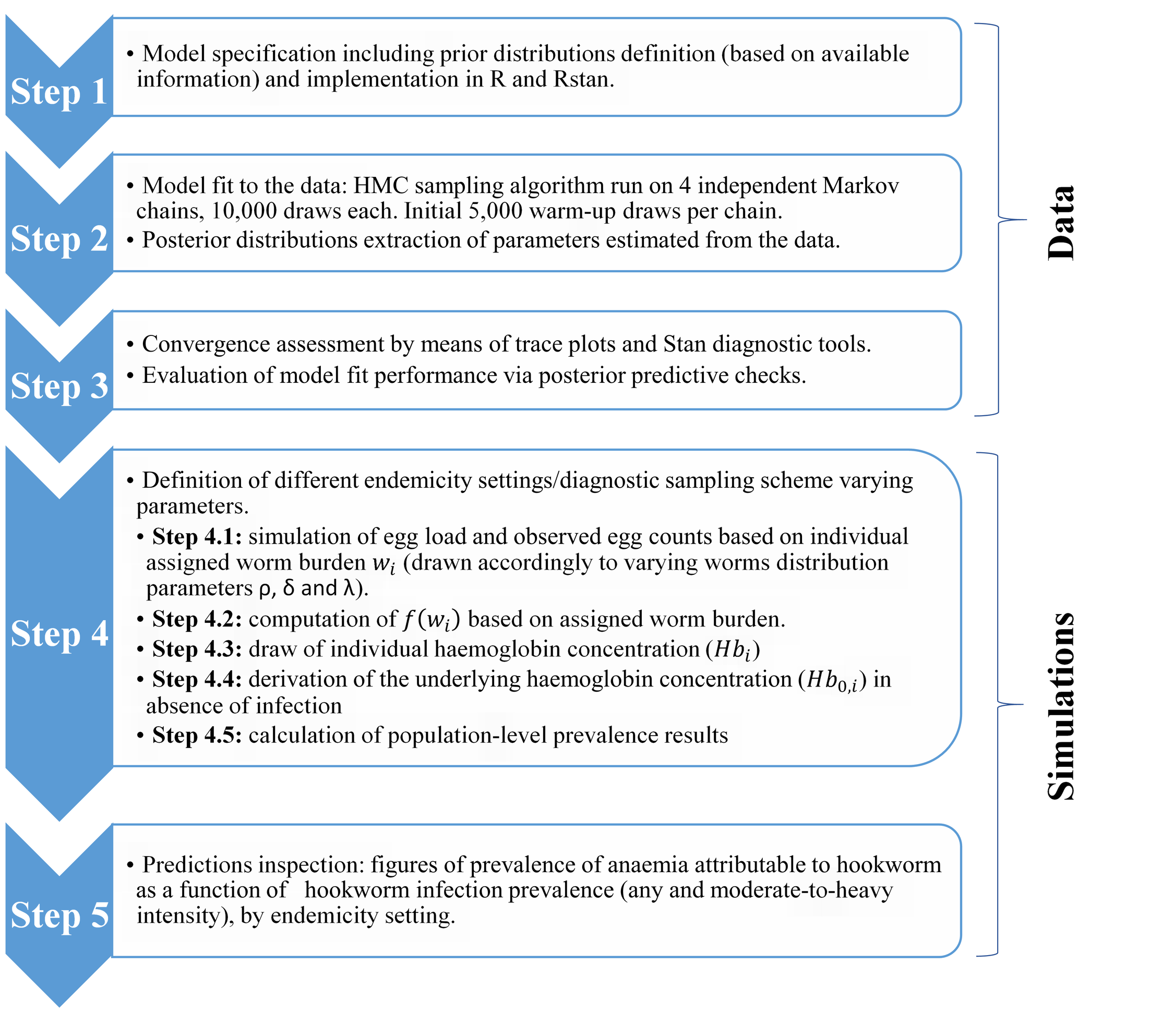

Supplement: S1 Fig — (TIF) [file pntd.0010279.s001.tif]

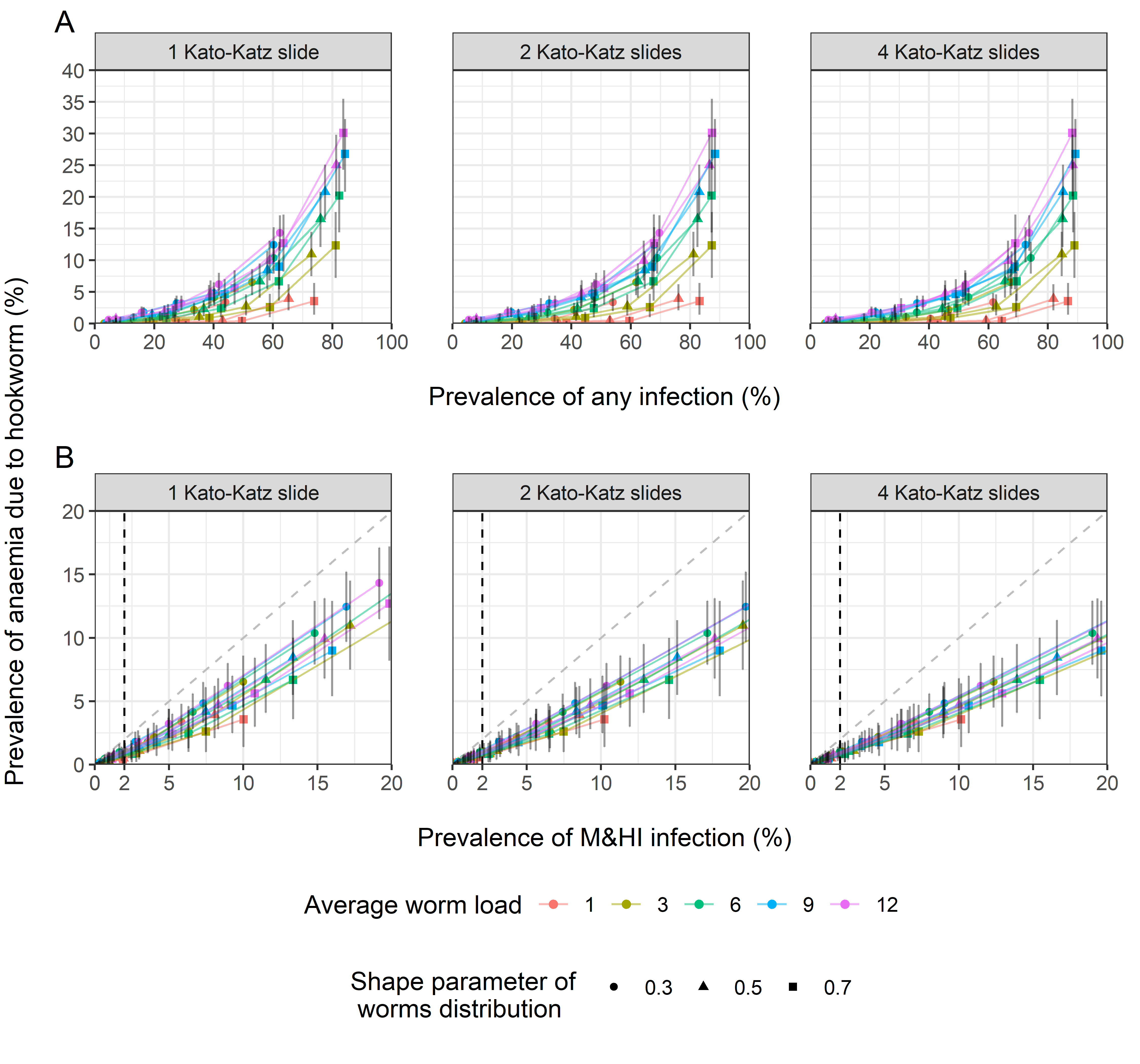

Supplement: S2 Fig — Predictions are displayed for different infection prevalence scenarios, defined by varying the probability ρ of having zero worms among the five values 0.1, 0.3, 0.5, 0.7, 0.9 (different points of the same colour), the population mean worm burden (different colours) and the shape parameter δ of worm distribution in infected individuals (different shapes). From left to right, the prevalence of any hookworm infection (A) and moderate-to-heavy intensity of infection (B) is based on 1, 2, 4 Kato-Katz slides, respectively. Each point (x, y) represents the mean predictions of infection prevalence (x-value) vs. hookworm-anaemia prevalence (y-value). The error band reports the 95% Confidence Interval (CI) of predicted hookworm-anaemia. Dashed vertical lines mark the WHO morbidity threshold of 2% moderate-to-heavy intensity prevalence. The bisector in panels (B) is added to visualise the robustness of the prevalence of moderate-to-heavy intensity of infection over the three sampling schemes. (TIF) [file pntd.0010279.s002.tif]
